# Evaluation of a remote biomarker capture system integrated with REDCap: A decentralized randomized trial

**DOI:** 10.1017/cts.2025.10160

**Published:** 2025-09-25

**Authors:** Jennifer Dahne, Amy E. Wahlquist, Jacob Kustanowitz, Juliana Hayden, Noelle Natale, John Clark

**Affiliations:** 1 Department of Psychiatry and Behavioral Sciences, University of ViennaMedical University of South Carolina, Charleston, SC, USA; 2 Hollings Cancer Center, Medical University of South Carolina, Charleston, SC, USA; 3 Center for Rural Health and Research, East Tennessee State University, Johnson City, TN, USA; 4 MountainPass Technology, Chevy Chase, MD, USA; 5 Information Solutions, Medical University of South Carolina, Charleston, SC, USA

**Keywords:** Decentralized trials, redcap, digital health, remote patient monitoring, clinical research informatics

## Abstract

**Introduction:**

Decentralized clinical trials (DCTs) are often hindered by challenges in remotely capturing biomarkers. To address this gap, we developed MyTrials, a mobile application integrated with REDCap, designed to facilitate the remote capture of biomarkers via Bluetooth-enabled remote patient monitoring (RPM) devices. The purpose of the present study was to evaluate the feasibility and acceptability of MyTrials among participants within a DCT design.

**Methods:**

In this four-arm randomized trial, 47 participants were allocated to receive zero, one, two, or three RPM devices. Participants were asked to use their devices once per week for a total of four weeks to remotely provide biomarkers via MyTrials. Feasibility was assessed using objective metrics of successful biomarker submission (i.e., valid device data accompanied by a video confirming participant identity) alongside the participant-reported Feasibility of Intervention Measure (FIM). Acceptability was evaluated via the Acceptability of Intervention Measure (AIM) and the System Usability Scale (SUS).

**Results:**

Among participants assigned at least one device, the successful biomarker submission rate was 74% across all study weeks. FIM and AIM scores exceeded prespecified feasibility benchmarks across all conditions except the zero-device condition. SUS scores consistently indicated high usability across all conditions (range: 77.29–94.29).

**Conclusions:**

The MyTrials platform is a feasible and acceptable solution for remote biomarker capture in DCTs. These findings support the potential of MyTrials to advance remote data collection in clinical research.

## Introduction

Decentralized clinical trials (DCTs; also referred to as distributed, remote, or virtual) can address longstanding challenges to clinical trials broadly by enhancing sample diversity, supporting treatment compliance, and promoting study retention [1–4]. Unfortunately, DCTs face an important methodological limitation: the need for biomarker collection. Biomarkers often serve as key trial outcomes and/or are used as adjuncts to patient-reported outcomes. Over the last decade, a multitude of Bluetooth-enabled remote patient monitoring (RPM) devices have become available which could allow researchers to remotely capture trial-relevant biomarkers. However, at least two translational research barriers currently limit widespread integration of RPM devices for remote biospecimen collection. First, existing RPM devices have been developed primarily for clinical utilization, not research utilization. This means that each device works with its own individual application (“app”) to initialize biomarker capture, store information, and share data with a clinical provider. Thus, there is need to integrate RPM devices with existing research data infrastructure. Second, individual RPM devices typically function independently, such that if an end-user uses multiple RPM devices, they need to use separate apps to capture data from each device. Thus, in addition to integrating RPM devices with existing research data infrastructure, there is need to facilitate deployment of multiple devices within any one trial.

One potential solution to the first translational barrier, the lack of integration with research infrastructure, is to leverage REDCap. REDCap is an online research data capture system that is freely available to research institutions and is currently in use across more than 7700 institutions in 160 countries [5]. RPM devices could be integrated with REDCap to facilitate the remote capture of biomarkers in DCTs. Our team previously demonstrated the feasibility and validity of this approach for one initial use case: remote expired air (i.e., breath) carbon monoxide (CO) capture [6]. To remotely capture CO, a key endpoint in smoking cessation clinical trials [7], we developed a participant-facing mobile app that allows individuals in DCTs to complete all self-report study assessments while also submitting a breath sample via a Bluetooth-enabled RPM device for CO analysis. The integration leverages an existing REDCap project, which was assigned a security token that allowed the app to use REDCap’s Application Programming Interface (API) to read the configuration of data collection instruments and write results into an individual study record within the project. Our team integrated the Bluetooth-enabled CO monitor with the participant-facing app by using the device manufacturer’s free and publicly available Software Development Kit (SDK). Instructions regarding use of the RPM device are all provided in the app, and the app captures a photo of the participant providing their breath sample, which is stored in the project’s REDCap database and can be used to verify participant identity.

To address the second translational barrier – facilitating the use of multiple RPM devices within a single trial – we expanded upon our initial approach. We developed a new participant-facing app, called “MyTrials,” and successfully integrated three additional commercially available Bluetooth-enabled RPM devices (thermometer, pulse oximeter, and blood pressure monitor) into the platform. These devices were selected for their broad relevance across diverse clinical contexts. MyTrials was also designed with flexibility in mind: additional RPM devices can be incorporated over time, and the platform can alternatively be used without any RPM devices to support the remote collection of patient-reported outcomes in a manner fully integrated with REDCap. The platform accommodates biomarker capture via one, two, or all three devices within a single REDCap project and includes video capture to verify participant identity during biospecimen collection.

In the present study, the primary objective was to describe the feasibility and the acceptability of MyTrials when deployed within a DCT design. Although MyTrials was developed primarily to support remote biomarker capture from one or more RPM devices in a manner integrated with REDCap, the platform can also be used to solely collect patient-reported outcomes. Thus, to evaluate the full range of use cases, we assessed feasibility and acceptability among participants using MyTrials with zero, one, two, or three RPM devices.

## Materials and methods

### Study design

This 4-arm randomized decentralized trial aimed to describe feasibility and acceptability of MyTrials when deployed with either zero, one, two, or three Bluetooth-enabled RPM devices. The study was approved by the institutional review board at the Medical University of South Carolina (Pro00117870). Participants provided electronic written informed consent.

### Participants

Inclusion and exclusion criteria were intentionally broad to maximize the generalizability of results to a wide population of participants who may enroll in DCTs. Inclusion criteria were: (1) age 18+, (2) currently own an iOS or Android smartphone capable of running MyTrials, (3) have access to an additional device with a webcam, such as a computer or tablet, to complete a synchronous baseline video call with research staff while also using MyTrials on their smartphone, (4) have a valid mailing address to receive mailed RPM devices, (5) report willingness to utilize an app to provide research data (response of “yes” on yes/no item), and (6) English fluency. Participants were excluded if another member of their household was currently participating in the study.

### Procedures

Participants were recruited online via social media platforms. Advertisements directed those interested to a REDCap online screener to preliminarily assess study eligibility. If preliminarily eligible, participants were scheduled to complete a synchronous, remote visit with study staff to complete informed consent. After completing consent, participants were randomized to either the zero, one, two, or three device condition, and then were mailed a package that contained the RPM devices consistent with their randomization assignment. Participants in the zero devices group received a mailing that solely contained information about the study. After receiving their mailed package, participants completed a synchronous baseline video visit with a member of the research team. The purpose of the baseline visit was to assist participants with downloading MyTrials and ensure that the technology was functional on the participant’s smartphone (study inclusion criterion). After the baseline session, participants were prompted via push notifications from MyTrials to submit their assigned biomarkers once per week for a total duration of four weeks. Participants were compensated via electronic payment $40 for completion of the synchronous baseline visit and $10 for successful completion of each of the four weekly assessments within 48 hours of being notified to submit their data, for a possible total of $80 for full participation.

### Randomization

Consented participants were randomized 1:1:1:1 to one of the four conditions (zero, one, two, or three RPM devices) utilizing a mixed block design. Individuals were stratified within the one and two RPM device groups to ensure balance between devices (thermometer, pulse oximeter, blood pressure monitor) and combinations of devices (thermometer+pulse oximeter, thermometer+blood pressure monitor, pulse oximeter+blood pressure monitor).

### MyTrials

**Figure 1.**
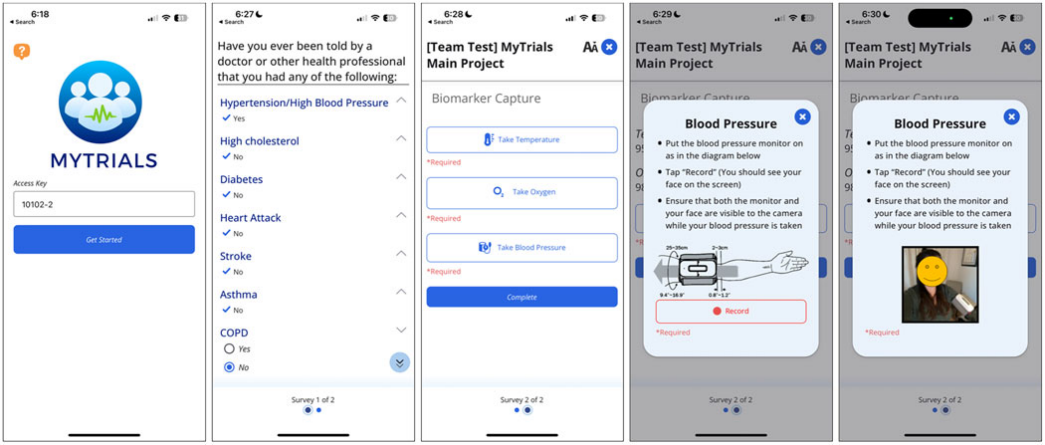
MyTrials screenshots (participant experience). Screenshots from the participant-facing MyTrials mobile application illustrate how users are guided to complete self-report assessments and submit biomarker data using Bluetooth-enabled remote patient monitoring devices. The app also captures a short video during biomarker collection to verify participant identity.

**Figure 2.**
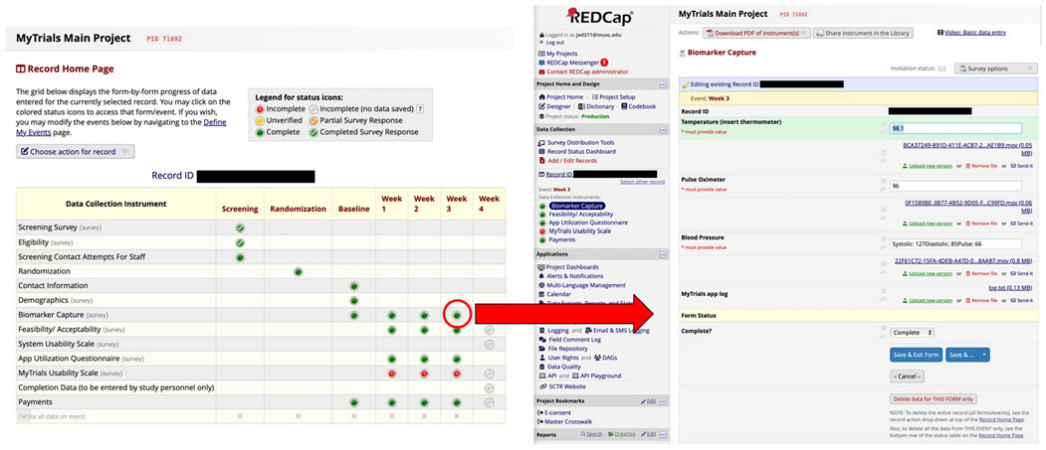
MyTrials screenshots (Researcher experience). Screenshots from the research team’s REDCap project demonstrate how MyTrials integrates remotely collected self-report and device-based biomarker data directly into study records. These examples show how data are organized and stored within REDCap, providing researchers with real-time access and verification capabilities.

MyTrials is available on both iOS and Android. As noted above, MyTrials works with REDCap [5] and leverages an existing REDCap project built by a research team as they would any other REDCap project. The project is assigned a security token which allows MyTrials to use REDCap’s API to read the configuration of data collection instruments and write results into an individual study record within the REDCap project. MyTrials walks the end-user through how to submit their biomarkers using their assigned RPM devices and also captures a video of the entire process of the end-user using their assigned RPM devices to provide their data. These video recordings can be used by the research team for identity verification. End-users can also complete any self-report assessments (i.e., patient-reported outcomes) within the MyTrials app, with all data (self-report and RPM device data) stored in the study’s REDCap project for a specific individual (i.e., record). See Figure 1 for screenshots of the MyTrials participant-facing app and Figure 2 for screenshots of a REDCap project that deployed MyTrials.

### Outcome assessments

At baseline, participants self-reported demographic information, including age, sex, race, ethnicity, educational attainment, annual household income, marital status, employment status, smartphone ownership, and physical and mental health comorbidities. Race and ethnicity options were defined by the study team consistent with Office of Management and Budget standards [8].

The primary outcomes for this study were feasibility and acceptability of MyTrials. Feasibility was assessed via both participant self-report and data captured from REDCap. At Week 4, within MyTrials, participants completed the Feasibility of Intervention Measure (FIM) [9], a 4-item measure scored on a 5-point Likert scale that measures the feasibility of an intervention by averaging the 4 items (ranges 1 to 5). Feasibility of biomarker submission was also assessed with objective indicators of success tracked in REDCap. At each assessment point, a biomarker was deemed successfully submitted if a valid value from the RPM device was entered into the REDCap project for the corresponding time point and was also accompanied by a video recording confirming that the intended participant was using the device to submit the biomarker.

Acceptability of MyTrials was assessed at Week 4 via participant self-report on the Acceptability of Intervention Measure (AIM) [9] and the System Usability Scale (SUS) [10]. Similar to the FIM, the AIM is a 4-item Likert scale measure that assesses acceptability of an intervention by averaging the 4 items in the instrument (ranges 1 to 5). The SUS is a validated 10-item self-report measure of acceptability; scores range from 0 to 100, and higher scores indicate greater usability.

### Sample size

The planned sample size for this study (*N* = 48; *n* = 12 per device group) was determined based on estimating 95% confidence intervals (CIs) for successful biomarker submission rates (operationalized as a valid submission from all assigned devices and corresponding videos confirming participant identity), with targeted goals of ≥80% submitted samples (95% CI ranging from 67% to 93% for those receiving at least one RPM device). The expected outcomes of feasibility (FIM ≥ 4 [9]) and acceptability (AIM ≥ 4 [9] and SUS ≥ 68 [10]) were determined *a priori* to demonstrate that MyTrials provided better than average feasibility, acceptability, and usability for users of the app. The planned sample size provided 80% power to detect effect sizes as small as 0.36, utilizing a one-sided *α* = 0.05 significance level.

### Statistical methods

Descriptive statistics such as means, standard deviations, frequencies, and percentages were used to describe both demographics and outcomes of feasibility (biomarker submission rates and FIM scores) and acceptability (AIM and SUS scores). These descriptive statistics were done both within and across randomization groups, depending on outcome. One-sided, one-sample *t*-tests were used to determine if scores on the FIM, AIM, and SUS were higher than the *a priori* determined benchmark values. For biomarker submission rates, the zero device group was not included, as no biomarker submissions were required for this group. Additionally, for biomarker submission rates, metrics across the 4 study weeks were summarized to provide overall feasibility across time.

## Results

### Participant characteristics

**Figure 3.**
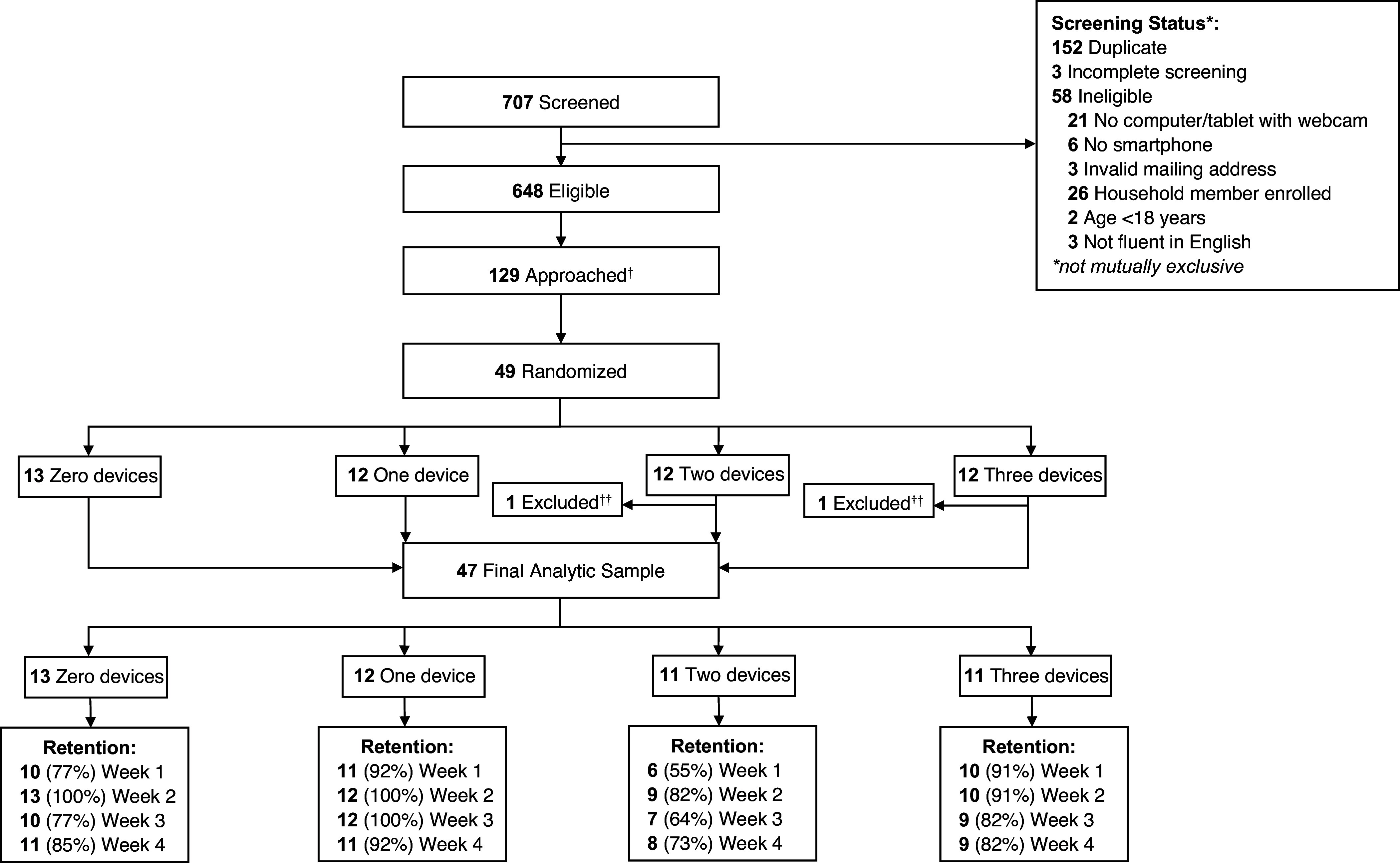
CONSORT diagram. ^†^Note that due to broad inclusion criteria and a small planned sample size, recruitment goals were met quickly, and therefore, not all eligible individuals were invited to provide informed consent. ^††^Participants were excluded post-randomization due to not owning an iOS or android smartphone capable of running MyTrials, consistent with *a priori* inclusion and exclusion criteria.

Participants were recruited from August 7, 2023, to February 5, 2024, and data collection was completed on March 11, 2024. Of 707 potential participants who completed preliminary study screening, 648 were preliminarily eligible, 129 were approached for participation, and 49 were randomized. Two participants were excluded post-randomization due to not owning an iOS or Android smartphone capable of running MyTrials, resulting in a final analytic sample of 47 participants (Figure 3). Due to the relatively small planned sample size and broad inclusion criteria, the planned sample size was met quickly after beginning trial accrual, and not all individuals who screened as preliminarily eligible were invited to complete informed consent. Among the 47 participants in the final analytic sample, 23 (49%) were female, 24 (51%) were male, and the mean (SD) age was 46.51 (15.44) years. The majority of participants (*n* = 28, 60%) were iPhone owners and the remaining participants (*n* = 19, 40%) were Android owners. Participant demographic information is outlined in Table 1.

**Table 1.** Participant characteristics

	All (*N* = 47)	Zero devices (*N* = 13)	One device (*N* = 12)	Two devices (*N* = 11)	Three devices (*N* = 11)
Age, mean (SD), y	46.51 (15.44)	45.38 (12.04)	45.08 (15.61)	49.18 (14.57)	46.73 (20.76)
Sex, No. (%)					
Female	23 (49)	7 (54)	3 (25)	6 (55)	7 (64)
Male	24 (51)	6 (46)	9 (75)	5 (45)	4 (36)
Race, No. (%)					
American Indian/Alaskan Native	1 (2)	0 (0)	0 (0)	1 (9)	0 (0)
Asian	9 (19)	3 (23)	3 (25)	2 (18)	1 (9)
Black or African American	10 (21)	3 (23)	4 (33)	1 (9)	2 (18)
Native Hawaiian or Other Pacific Islander	2 (4)	0 (0)	1 (8)	0 (0)	1 (9)
White	21 (45)	5 (38)	4 (33)	5 (45)	7 (64)
Multiracial	2 (4)	2 (15)	0 (0)	0 (0)	0 (0)
Other	2 (4)	0 (0)	0 (0)	2 (18)	0 (0)
Ethnicity					
Hispanic/Latinx, No. (%)	2 (4)	0 (0)	1 (8)	1 (9)	0 (0)
Education, No. (%)					
≤ High School diploma	3 (6)	0 (0)	2 (17)	1 (9)	0 (0)
> High School diploma	44 (94)	13 (100)	10 (83)	10 (91)	11 (100)
Annual household income, No. (%)					
< $50k	14 (30)	5 (38)	7 (58)	0 (0)	2 (18)
≥ $50k	32 (68)	8 (62)	5 (42)	10 (91)	9 (82)
Unknown/Refused	1 (2)	0 (0)	0 (0)	1 (9)	0 (0)
Marital status, No. (%)					
Married	17 (36)	5 (38)	2 (17)	4 (36)	6 (55)
Divorced or separated	8 (17)	3 (23)	3 (25)	1 (9)	1 (9)
Single and never been married	15 (32)	3 (23)	5 (42)	4 (36)	3 (27)
Member of an unmarried couple	7 (15)	2 (15)	2 (17)	2 (18)	1 (9)
Employment status, No. (%)					
Unemployed	4 (9)	1 (8)	3 (25)	0 (0)	0 (0)
Employed full time	24 (51)	6 (46)	5 (42)	7 (64)	6 (55)
Employed part time	7 (15)	4 (31)	1 (8)	1 (9)	1 (9)
Retired	7 (15)	0 (0)	1 (8)	3 (27)	3 (27)
Student	3 (6)	1 (8)	1 (8)	0 (0)	1 (9)
Other	2 (4)	1 (8)	1 (8)	0 (0)	0 (0)
Type of smartphone, No. (%)					
iPhone	28 (60)	8 (62)	8 (67)	7 (64)	5 (45)
Android	19 (40)	5 (38)	4 (33)	4 (36)	6 (55)
Ever been told by a health care professional that you had a certain condition, No, (%)					
Hypertension	12 (26)	2 (15)	4 (33)	2 (18)	4 (36)
High cholesterol	13 (28)	4 (31)	4 (33)	1 (9)	4 (36)
Diabetes	10 (21)	3 (23)	2 (17)	2 (18)	3 (27)
Heart attack	1 (2)	0 (0)	1 (8)	0 (0)	0 (0)
Asthma	5 (11)	3 (23)	0 (0)	2 (18)	0 (0)
Cancer	3 (6)	1 (8)	0 (0)	0 (0)	2 (18)
Kidney disease	1 (2)	1 (8)	0 (0)	0 (0)	0 (0)
Depression	12 (26)	6 (46)	4 (33)	1 (9)	1 (9)
Anxiety disorder	8 (17)	4 (31)	2 (17)	1 (9)	1 (9)
Other mental health disorder	2 (4)	2 (15)	0 (0)	0 (0)	0 (0)

### Feasibility

**Figure 4.**
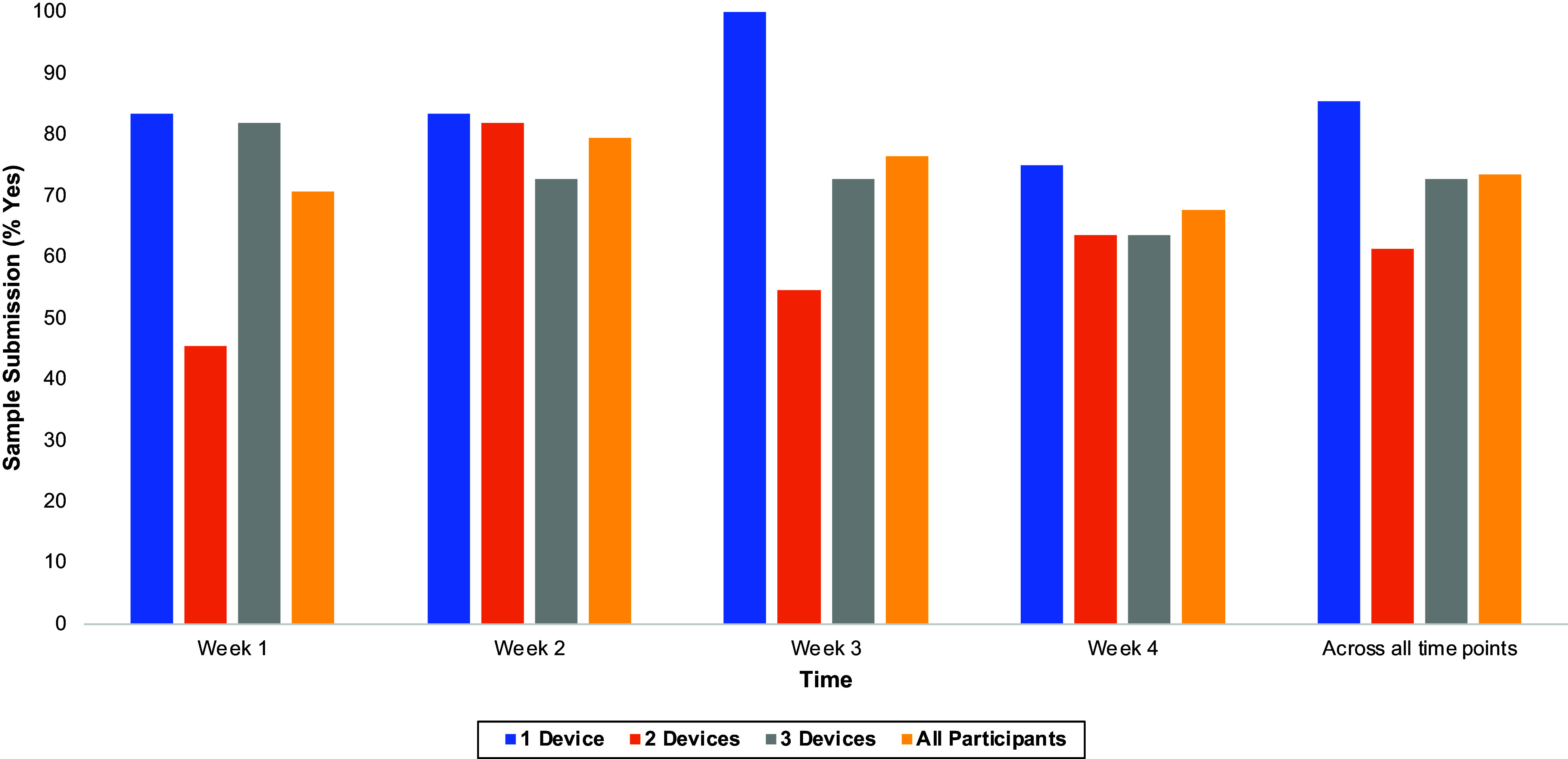
Successful sample submission rate as a function of randomization group. Successful sample submission defined as a valid value from the RPM entered into the REDCap project for the corresponding time point accompanied by a video recording confirming that the intended participant was using the device to submit the biomarker.

Biomarker submission rates were relatively high across all device groups throughout the study, with 71% of individuals (24/34 receiving at least one device) submitting a valid value with identity-confirmed video at Week 1, 79% (27/34) at Week 2, 76% (26/34) at Week 3, 68% (23/34) at Week 4, and 74% of all possible submissions (100/136: 34 individuals across 4 time points) with confirmed identity and valid values (Figure 4). The one device group had the highest submission rates (ranging from 75% to 100%, based on week), followed by the three device group (64% to 82%), and the two device group (45% to 82%) (Figure 4, Table 2).

**Table 2.** My trials feasibility and acceptability

	All (*N* = 47)*	Zero devices (*N* = 13)	One device (*N* = 12)	Two devices (*N* = 11)	Three devices (*N* = 11)
**Successful sample submission**					
Week 1, No. (%)^†^	24 (71)	N/A	10 (83)	5 (45)	9 (82)
Week 2, No. (%)^†^	27 (79)	N/A	10 (83)	9 (82)	8 (73)
Week 3, No. (%)^†^	26 (76)	N/A	12 (100)	6 (55)	8 (73)
Week 4, No. (%)^†^	23 (68)	N/A	9 (75)	7 (64)	7 (64)
Across all timepoints, No. (%)	100 (74)	N/A	41 (85)	27 (61)	32 (73)
**Participant-reported feasibility and acceptability**					
FIM, mean (SD)	4.61 (0.78)	4.25 (1.11)	4.80 (0.31)	4.96 (0.09)	4.58 (0.83)
AIM, mean (SD)	4.44 (1.05)	3.71 (1.50)	4.86 (0.45)	4.93 (0.19)	4.53 (0.75)
SUS, mean (SD)	85.32 (18.59)	77.29 (25.26)	86.14 (15.55)	94.29 (7.46)	88.06 (15.60)

Successful sample submission defined as a valid value from the RPM entered into the REDCap project for the corresponding time point accompanied by a video recording confirming that the intended participant was using the device to submit the biomarker. FIM = Feasibility of Intervention Measure; AIM = Acceptability of Intervention Measure; SUS = System Usability Scale. *The zero-device group was not included in descriptive biomarker submission outcomes because participants randomized to that group were not asked to submit biomarkers. Thus, the denominator for this column is *N* = 34. ^†^Data are provided as the number of samples successfully submitted divided by the number due for submission.

Feasibility at Week 4 based on the FIM total score (average of 4 items) was completed by all individuals, inclusive of those in the zero device group. The mean (SD) FIM total score for all groups exceeded the expected benchmark of 4 for feasibility: 4.61(0.78), *p* < 0.001. When considering each group separately, all groups except the zero device group also exceeded the expected benchmark value of 4 for feasibility: zero device = 4.25(1.11), *p* = 0.2; one device = 4.80(0.31), *p* < 0.001; two device = 4.96(0.09), *p* < 0.001; three device = 4.58(0.83), *p* = 0.03 (Table 2).

### Acceptability

Similar to feasibility, acceptability was measured by the AIM total score (average of 4 items) at Week 4 for all participants, regardless of group. Across all participants, the total AIM score exceeded the expected benchmark of 4 for acceptability: 4.44 (1.05), *p* = 0.006. For all groups that received a device, the mean(SD) benchmark for acceptability was achieved: one device = 4.86 (0.45), *p* < 0.001; two device = 4.93 (0.19), *p* < 0.001; three device = 4.53 (0.75), *p* = 0.03. The zero device group had a slightly lower average [3.71 (1.50), *p* = 0.7] for this outcome (Table 2).

The mean(SD) SUS total scores across all device groups [85.32 (18.59)] was much higher than the expected benchmark value of 68 (*p* < 0.001), indicating high usability across groups. All individual device group comparisons, with the exception of the zero device group, were also significantly higher than the benchmark value: one device = 86.14 (15.55), *p* = 0.001; two device = 94.29 (7.46), *p* < 0.001; three device = 88.06 (15.60), *p* = 0.002. The zero device group had a mean point estimate higher than the benchmark value but a wide range of responses: 77.29 (25.26), *p* = 0.1 (Table 2).

## Discussion

In this DCT, we evaluated the feasibility and acceptability of MyTrials for supporting remote biomarker capture. Among this adult sample, for groups that received at least one RPM device, MyTrials generally exceeded prespecified feasibility and acceptability benchmarks. Regarding the successful sample submission (valid data from devices and video confirming participant identity) feasibility metric, the one device condition most consistently exceeded the prespecified feasibility benchmark (80%), with this benchmark exceeded at Weeks 1, 2, and 3. However, the two and three device conditions inconsistently exceeded this feasibility benchmark (only at Week 2 for the two device condition and Week 1 for the three device condition). This suggests that additional refinements may be needed, particularly when the app is deployed to support collection of multiple biomarkers via different RPM devices, to further promote participant compliance.

Participant self-reported product feasibility and acceptability were strong across all groups assigned RPM devices, and these outcomes consistently exceeded prespecified benchmarks for considering MyTrials feasible and acceptable. Regarding potential areas for future improvement, participants in the zero device group rated MyTrials the lowest across measures of feasibility, acceptability, and usability. This could in part be due to a disappointment effect given that these participants enrolled in a DCT to provide biomarkers remotely and were assigned to a condition in which they were not asked to provide biomarkers. MyTrials fills a unique niche specifically to support remote biomarker capture in a manner integrated with REDCap. Other products (e.g., the MyCap app [11]) currently exist to facilitate remote capture of patient-reported outcomes via a mobile app integrated with REDCap. Thus, MyTrials may be most useful for studies that need a feasible and acceptable platform that functions in combination with REDCap to remotely capture biomarkers. MyTrials may be less appropriate for studies that solely capture patient-reported outcomes.

Platforms, like MyTrials, that support remote biomarker capture in a manner integrated with existing research infrastructure, can help to address lingering data quality and integrity concerns related to the conduct of DCTs [12]. By facilitating participant identity verification and leveraging existing data quality checks available within REDCap, researchers who use MyTrials can ensure that data captured in their DCT is valid. Importantly, in DCT designs, biomarkers may be captured remotely via methods other than RPM devices. For example, within decentralized tobacco trials, many investigators remotely capture saliva, either via rapid point-of-contact tests or mailed sample return, to assay for cotinine, a nicotine metabolite [7]. To continue to mitigate data quality and integrity risks as relevant to DCTs, the MyTrials platform can be further expanded both to support additional RPM device integration as well as to support other methods of remote biomarker capture, including point-of-contact testing and mailed sample return.

### Limitations

This study had a few limitations. First, all participants were required to own an iOS or Android device capable of running MyTrials. Generalizability to individuals with lower levels of digital proficiency may be limited. Second, while randomization was stratified to ensure balance between devices and combinations of devices, there were relatively few participants within each unique combination of devices group. Thus, it is difficult to determine whether feasibility and acceptability outcomes are driven by specific devices or by more generally the number of devices the participant was asked to use. Finally, feasibility and acceptability were assessed only through four weeks, and outcomes relied exclusively on quantitative measures. Longer-term follow-up and incorporation of qualitative feedback could have yielded additional insight into challenges with sustained engagement and usability (e.g., device pairing, video recording), and better informed future workflow refinements.

## Conclusions

Our findings support that MyTrials is generally a feasible and acceptable platform to remotely capture biomarkers via Bluetooth-enabled RPM devices in a manner integrated with REDCap. Additional future product refinements may help to bolster feasibility and acceptability when participants are asked to use more than one RPM device. Thus, MyTrials may provide a useful platform for researchers conducting DCTs who seek to remotely capture biomarkers.
